# Extending health equity to people with moderate and mild hemophilia A: revisiting the HAVEN 6 trial

**DOI:** 10.1016/j.rpth.2024.102648

**Published:** 2024-11-29

**Authors:** Cedric Hermans, Michiel Coppens, Giuliana Ventriglia, Gavin Ling, Michaela Lehle, Steven W. Pipe

**Affiliations:** 1 Division of Haematology, Cliniques Universitaires Saint-Luc, Université Catholique de Louvain (UCLouvain), Brussels, Belgium; 2 Department of Vascular Medicine, Amsterdam University Medical Center, University of Amsterdam, Amsterdam, The Netherlands; 3 Amsterdam Cardiovascular Sciences, Pulmonary Hypertension & Thrombosis, Amsterdam, The Netherlands; 4 Product Development Clinical Science, Oncology & Haematology, F. Hoffmann-La Roche Ltd, Basel, Switzerland; 5 Product Development Clinical Science, Haematology, Roche Products Ltd, London, United Kingdom; 6 Department of Pediatrics and Pathology, University of Michigan, Ann Arbor, Michigan, USA

**Keywords:** cost of illness, factor VIII, female, health equity, hemophilia A

## Abstract

Congenital hemophilia A (HA) disease severity has traditionally been categorized according to intrinsic factor (F)VIII levels, with <1% of normal indicating severe HA, 1% to 5% moderate HA, and 6% to 40% mild HA. However, mounting evidence illustrates considerable variability in bleeding phenotype regardless of FVIII level. Despite treatment advances, people with moderate or mild HA may be neglected, as treatment guidelines and established norms focus on FVIII levels, and many clinical trials do not include people with FVIII > 1%. Data from the HAVEN 6 trial demonstrated that people with moderate or mild HA, for whom prophylaxis was warranted by the treating physician’s judgment, experienced a clear clinical benefit from receiving emicizumab prophylaxis. A shift in treatment paradigms to incorporate clinical phenotypes alongside FVIII levels should be encouraged. This change in practice would allow treaters to extend health equity to people with moderate or mild HA.

## 1 Introduction

Congenital hemophilia A (HA) is an inherited, X-linked bleeding disorder characterized by a deficiency of coagulation factor (F)VIII [1]. Historically, HA severity has been defined according to intrinsic FVIII activity, with <1% of normal levels corresponding to severe HA, 1% to 5% of normal indicating moderate HA, and 6% to 40% of normal indicating mild HA; severity of bleeding episodes are expected to correlate with this categorization [1]. However, in recent years, it has been more broadly recognized that plasma FVIII activity does not uniformly correlate with bleeding phenotype, and by reserving prophylactic care for people with severe HA, we may be neglecting and undertreating people with moderate or mild HA [2].

Findings from numerous studies suggest that non-severe HA carries a significant disease burden, including repeated bleeding, permanent joint damage, pain, and quality of life impairment [[3], [4], [5], [6]]. Women and girls with FVIII deficiency face additional disease burden, with the potential for heavy menstrual bleeding and increased risks during pregnancy and birth, such as bleeding from invasive procedures and serious postpartum bleeding events [7,8].

The latest edition of the World Federation of Hemophilia Guidelines acknowledges the change in perspective that has come with the rapid progression in the therapeutic landscape for HA, as well as the need for prophylaxis in people with non-severe HA with a severe bleeding phenotype [1]. However, dedicated data on treatments for this population have been sparse.

Emicizumab is a recombinant, humanized, bispecific monoclonal antibody that mimics the function of activated FVIII by bridging activated FIX and FX, thereby improving hemostasis in people with HA [9]. HAVEN 6 (NCT04158648) is a multicenter, open-label, single-arm phase 3 study and the first emicizumab trial dedicated to people with moderate or mild HA [10]. Eligible participants warranted prophylactic treatment based on their treating physician’s assessment. The primary analysis of the HAVEN 6 trial demonstrated no new safety signals in this population and clinically meaningful bleed control irrespective of disease severity, dosing regimen, or prior treatment. In light of these results, and given the amplifying calls to strive for health equity among males and females with moderate or mild HA [11], this article will revisit the HAVEN 6 trial, examine the clinical implications, and explore future directions for treating people with moderate or mild HA.

## 2 Bleeding Phenotype and The Need for Prophylaxis Among People With Moderate Or Mild HA

Although treatments for HA have primarily been targeted at people with the severe form of the disease according to FVIII activity levels (or people with FVIII inhibitors), it is now increasingly recognized that many people with moderate or mild HA may have a bleeding phenotype that warrants prophylaxis. In HAVEN 6, the reasons reported by the investigators for participants requiring prophylaxis comprised a history of frequent bleeding (57% of participants), history of frequent joint bleeding (44%), history of severe bleeding (21%), prevention of traumatic bleeds (13%), or other (7%) [10]. These reasons were supported by the participants’ bleeds in the 24 weeks prior to receiving emicizumab, at which time 51% were receiving prophylactic treatment and 49% were receiving episodic treatment; the model-based annualized bleed rate (ABR) for all bleeds during this 24-week time period was 10.1 (estimated using negative binomial regression; 95% CI, 6.93-14.76) [10]. Similarly, in the PROBE study, 9.9% of males with mild hemophilia, 31.4% of males with moderate hemophilia, and 4.8% of females with hemophilia had an ABR of >10, despite 11.8%, 43.3%, and 2.4% of these groups, respectively, receiving regular or intermittent prophylaxis [6].

FVIII activity levels are influenced by *F8* genotype, X chromosome inactivation in females, and FVIII pharmacokinetics, which in turn is shaped by factors such as age, body mass index, plasma von Willebrand factor antigen binding and clearance, and blood type [[12], [13], [14], [15], [16]]. Any of these components can contribute to deficient hemostasis in people with moderate or mild HA. However, FVIII activity levels are only one part of the story; a number of other genetic and nongenetic factors are believed to influence bleeding phenotype, illustrating the overly simplistic nature of treating according to FVIII level categorization (Figure).

**Figure:**
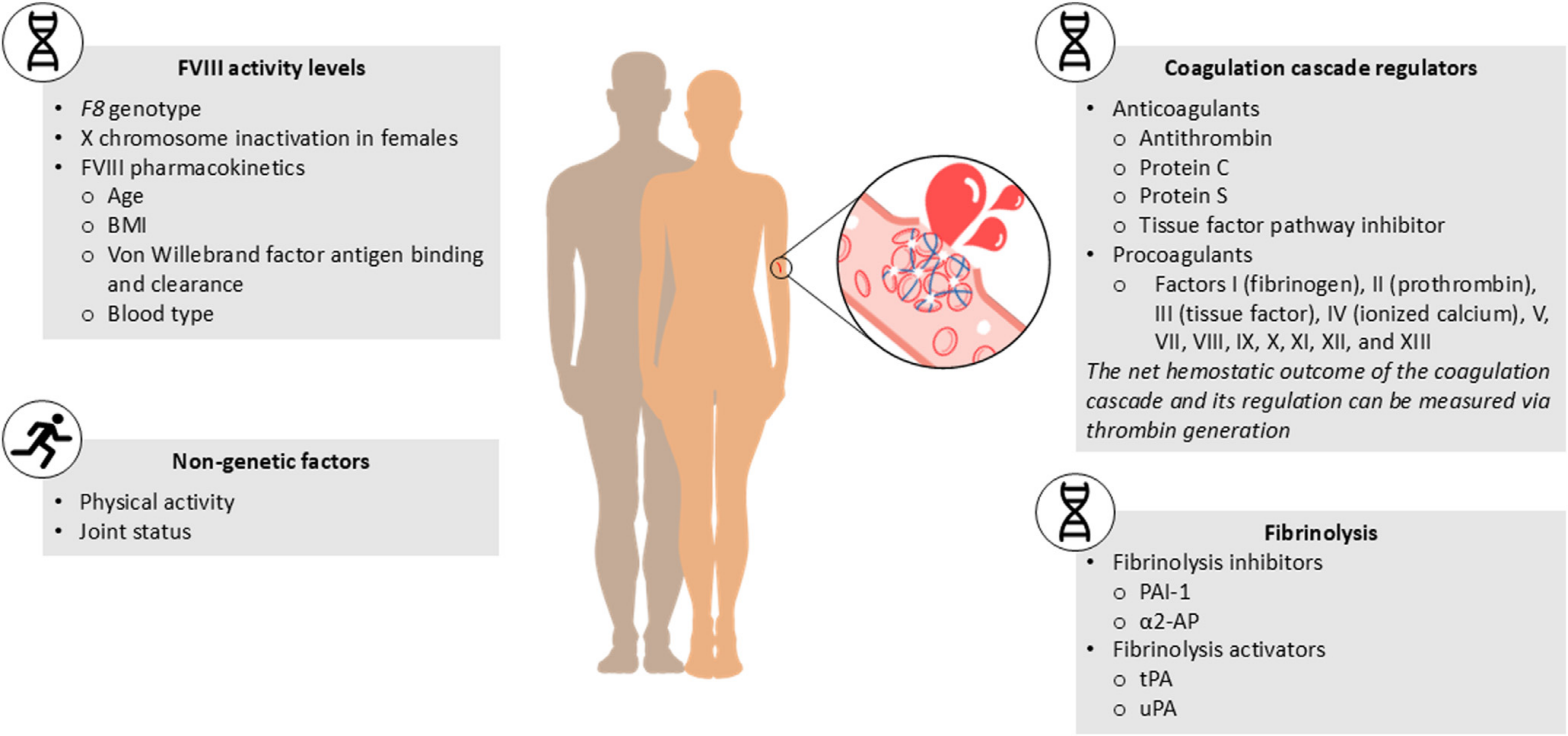
Factors contributing to bleeding phenotype. α2-AP, α2-antiplasmin; BMI, body mass index; FVIII, factor VIII; PAI-1, plasminogen activator inhibitor-1; tPA, tissue plasminogen activator; uPA, urokinase-type plasminogen activator.

Data from the HAVEN 6 trial demonstrated that people with moderate or mild HA, for whom prophylaxis was warranted by the treating physician’s judgment, experienced a clear clinical benefit from receiving emicizumab prophylaxis despite intrinsic FVIII levels above 1%. After a median follow-up of 55.6 weeks on emicizumab, the model-based ABR was 2.3 (95% CI, 1.67-3.12) for all bleeds and 0.9 (95% CI, 0.55-1.52) for treated bleeds [10], a considerable decrease from the prestudy model-based ABR of 10.1 (95% CI, 6.93-14.76).

## 3 Prophylaxis in Women and Girls with Moderate or Mild HA

Three female participants were included in HAVEN 6: 2 with mild HA and 1 with moderate HA [17]. Reasons given for warranting prophylaxis were a history of frequent bleeding and joint bleeding in 1 participant with mild HA, a history of frequent bleeding in the other participant with mild HA, and a history of severe bleeding in the participant with moderate HA. While receiving emicizumab, their ABRs for all bleeds were 0, 2.8, and 11.6, respectively. The participant with an on-study all-bleed ABR of 11.6 had an estimated all-bleed ABR prior to study entry of 208.6 (further details are reported in Hermans et al. [17]). The female participants in HAVEN 6 were asked to report their menstrual bleeding and menstruation-related quality of life; 2 of the 3 participants completed this questionnaire prior to emicizumab initiation, and both, including one with a history of heavy menstrual bleeding, indicated improvements in menstrual health by 9 weeks after initiating emicizumab, which were maintained until the latest timepoint evaluated [17]. Post menarche, women and girls with HA often experience heavy menstrual bleeding, and its severity across bleeding disorders does not show a linear correlation with FVIII level [18]. Consequently, heavy menstrual bleeding should be considered as an additional clinical need for people with moderate or mild HA who menstruate. Although interpretation of the data is limited by the small sample size, the improvements observed suggest that people with HA with heavy menstrual bleeding or other menstruation-related impairments in quality of life may benefit from prophylaxis [17].

The ATHN 7 observational study included 3 female participants with HA receiving emicizumab prophylaxis [19]. Two of these had severe HA and no history of FVIII inhibitors; the third had moderate HA and a history of low-titer FVIII inhibitors. The participant with moderate HA was 5 years old at study entry and had >150 days of prior exposure to FVIII. She experienced no bleeds while on emicizumab (duration of exposure, 94.4 weeks) [19].

## 4 Pharmacokinetics, Pharmacodynamics, and Safety of Emicizumab in People with Moderate or Mild HA

There is a hypothesis that an additive effect of FVIII and emicizumab could increase thrombotic risk in the non-severe HA population. An *in vitro* study showed an additive effect of emicizumab with FVIII but found that this effect diminished with increasing FVIII levels [20]. Based on preclinical data on the mechanism of action of emicizumab, the dose of emicizumab used in HAVEN 6 is the standard dose that has been used for individuals with severe HA or HA with FVIII inhibitors. The use of a lower dose of emicizumab was not clinically tested in people with mild/moderate HA. As the study population warranted prophylaxis, the intent was to provide an efficacious dose for prophylaxis.

Pharmacodynamic analysis from HAVEN 6, including measures of FVIII-like activity and thrombin generation, aligns with preclinical predictions and indicates that the emicizumab dose used did not exceed expected levels, supporting the safety of using full emicizumab dosing regimens [21]. Although a higher plasma emicizumab concentration was observed in HAVEN 6 than in previous HAVEN trials in people with severe HA, there is currently no evidence to suggest that this was due to endogenous FVIII activity in people with moderate or mild HA [10].

In HAVEN 6, 1 thrombotic event was observed over a median safety follow-up of 55.6 weeks (range, 8.7-89.9) [10]. This was a Grade 1 thrombosed hemorrhoid, classified as a thrombotic event according to the Medical Dictionary for Regulatory Activities system. The event occurred in a 49-year-old white male after >300 days of study and was considered unrelated to emicizumab due to the presence of obstipation, hemorrhoids, and a history of sigmoid diverticulitis. These clinical data added to preclinical data support the evidence that endogenous FVIII activity at the level of moderate or mild HA does not work in concordance with emicizumab to induce a hypercoagulable state [10,22]. Furthermore, during HAVEN 6, 31.4% of the participants with moderate HA and 38.1% of the participants with mild HA received on-demand FVIII treatment for a breakthrough bleed, with no associated thrombotic events or thrombotic microangiopathies [23].

## 5 Achieving Treatment and Health Equity for People with Moderate or Mild HA

Prophylactic care should be considered for any person with moderate or mild HA who has a disease burden that prophylaxis may be expected to improve according to the patient’s reported experience and the treating physician’s judgment [1]. With progress in approved and investigational treatment options for HA, including emicizumab, extended half-life FVIII, rebalancing agents, and gene therapies, near-normal hemostasis has become a realistic treatment goal and a common aim in the hemophilia community. We expect the achievement of this goal to result in an ABR of 0 or near 0, a quality of life similar to people without HA, and a hemophilia-free mind with all the freedoms that encompasses [24]. However, the opportunity to use the expanded range of therapeutic advancements to offer suitable prophylactic options for people with moderate or mild HA is currently underexploited [2,11].

Fortunately, we are now beginning to see increased inclusion of people with moderate or mild HA in trials of new therapies for HA, although this is not being consistently implemented, and people with moderate or mild HA are still often excluded. Furthermore, while many clinical trials allow for the inclusion of women and girls with HA, few are enrolled. To address the unmet needs of this population, further data on the issues specific to women and girls are essential, with the implementation of female-specific bleed endpoints and tools and the encouragement of clinicians to consider discussing trial enrollment with their female patients. To expedite the shift toward equitable trial design, we would welcome the development of guidelines recommending how to conduct inclusive clinical trials within HA, containing advice on inclusion criteria, endpoints, and assessment tools that address the needs of all people with HA. We suggest that these guidelines be developed in close collaboration with a range of stakeholders within the HA community, including people with moderate or mild HA and women and girls with HA. We do not anticipate any statistical limitations would be posed by an inclusive trial design, with the exception of small sample sizes for endpoints that are specifically focused on women and girls. As access to novel treatments is expanded, the publication of clinical trials and real-world data on treatment outcomes from the whole spectrum of people with HA would support evidence-based treatment decision-making.

## 6 Re-evaluating Treatment Paradigms for HA

As this forum article has explored, the bleeding phenotype of people with HA is impacted by various factors besides FVIII activity levels. The needs of people with moderate or mild HA are frequently underestimated and overlooked, and those with a bleeding tendency stand to benefit greatly from prophylaxis. Thus, it is time to reevaluate treatment paradigms for HA; a combined complementary laboratory (FVIII levels) and clinical (bleeding phenotype) assessment could be implemented when evaluating people with HA. Indeed, this has already been recommended by the European Hematology Association, which suggested distinguishing individuals with a pathological bleeding tendency based on a standardized quantitative assessment of bleeding history [25]. Here, we further argue that clinical phenotype is the most important factor when considering treatment and determining whether individuals will benefit from prophylaxis.

Improving care in this population demands further education on the implications of non-severe HA. People with moderate or mild HA should receive regular follow-up assessments, including joint health monitoring and access to innovative treatment options. Prophylaxis in a person with moderate or mild HA should be started at an early age to maximize bleed prevention and joint protection [2]. The criteria to consider when evaluating people with moderate or mild HA to determine candidacy for prophylaxis are highly variable from country to country, as these depend on the resources available and economic restrictions of each country’s healthcare system; it is, therefore, challenging to reach an international consensus on how to select individuals for prophylaxis. Nevertheless, the following proposed criteria have been described by Castaman et al. [2]: risk factors for bleeding; presence of comorbidities; early signs of joint degeneration; desired lifestyle and daily activities that would increase bleeding risk; symptoms of a severe bleeding phenotype, including experiencing the first bleed before 6 months of age, first joint bleed before 2 years of age, having spontaneous bleeds in critical sites, or having 2 or more spontaneous bleeds per year; and a history of severe, potentially life-threatening, bleeds. The shared decision-making process should be revisited regularly throughout the lifetime of the individual to ensure the lifelong burden of treatment is balanced with the lifelong burden of disease. It is crucial that, as we continue through this new era of rapid treatment advancements and novel therapies to treat HA, people with non-severe HA are not left behind.

## 7 Conclusions

With advancements in therapy, the population of people with HA who would benefit from prophylaxis has changed. This forum paper has explored the evidence supporting prophylaxis for people with moderate or mild HA for whom it is clinically warranted, with a focus on the HAVEN 6 trial dedicated to the assessment of emicizumab in people with moderate or mild HA, including women and girls. We urge the community to enact a change in treatment paradigms and guidelines for HA to replace FVIII activity with clinical phenotype as the most important criterion for determining the need for prophylactic care. Only by prioritizing the lived experience of people with HA can we achieve health equity for all.
